# Phase-Shifting Structured Illumination with a Polarization-Encoded Metasurface

**DOI:** 10.1021/acs.nanolett.5c02789

**Published:** 2025-07-17

**Authors:** Linzhi Yu, Jesse Pietila, Haobijam Johnson Singh, Humeyra Caglayan

**Affiliations:** † Department of Physics, 201768 Tampere University, 33720 Tampere, Finland; ‡ Department of Electrical Engineering and Eindhoven Hendrik Casimir Institute, 7840 Eindhoven University of Technology, 5600 MB Eindhoven, Netherlands

**Keywords:** metasurfaces, phase-shifting structured illumination, fringe projection profilometry, 3D measurement

## Abstract

Phase-shifting structured illumination is a powerful technique used across diverse imaging modalities including 3D surface measurement, quantitative phase imaging, and super-resolution microscopy. However, conventional implementations often rely on mechanically or optoelectronically driven complex systems, limiting the compactness, stability, and integration. Here, we present a polarization-controlled dielectric metasurface that generates phase-shifting fringe patterns in the visible spectrum, enabling compact and robust structured light projection. The metasurface encodes distinct phase gratings for orthogonal polarizations, producing fringe patterns with lateral displacements that vary with the transmitted polarization. We experimentally demonstrate high-quality fringe generation and apply the structured illumination in a fringe projection profilometry system for the 3D surface measurement of different objects. The metasurface integrates multiple phase-shifting steps into a single static device, offering a millimeter-scale footprint and compatibility with polarization multiplexing. This approach introduces a compact, passive solution for structured light generation with broad potential in optical metrology and computational imaging.

## Graphical abstract

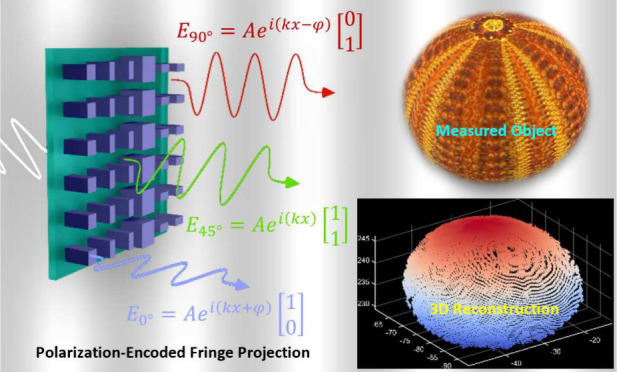

Phase-shifting structured illumination plays a key role in a wide range of optical imaging applications,^1^ including three-dimensional (3D) surface measurement,^2,3^ quantitative phase imaging,^4,5^ and super-resolution microscopy.^6,7^ In these modalities, sinusoidal fringe patterns with controlled phase offsets are projected onto objects and the resulting intensity maps are processed to reconstruct surface topography, optical path length variations, or fine spatial features beyond the diffraction limit. A key requirement for such systems is the ability to generate high-quality fringe patterns with tunable phase shifts. Traditional methods achieve phase shifting by mechanically moving gratings^8,9^ or adjusting the relative optical path length between interfering beams.^10,11^ While effective, these approaches suffer from limited stability, slow response times, and poor scalability. More recently, active spatial modulation devices, such as spatial light modulators,^12,13^ digital micromirror devices,^14,15^ and acousto-optic deflectors,^16,17^ have been introduced to enhance programmability and speed. However, their relatively large pixel sizes and bulky optical setups hinder their integration into compact, high-resolution systems.

Metasurfaces offer a promising path forward. These ultrathin optical elements, composed of subwavelength-spaced meta-atoms, enable spatially varying modulation of light’s amplitude, phase, and polarization in a single, nanostructured layer.^18–30^ Plasmonic metasurfaces have demonstrated phase-shifting structured light via surface plasmon interference^31–34^ but suffer from high optical loss, particularly in the visible range.^35^ Based on high-index, low-loss materials, dielectric metasurfaces have emerged as a superior alternative.^36^ However, existing designs typically generate fixed patterns^37^ or require complex co-modulation of both the illumination and detection pathways,^38^ limiting their flexibility and complicating integration into compact or adaptive imaging systems.

In this work, we present a polarization-controlled dielectric metasurface capable of generating high-quality phase-shifting structured illumination in the visible spectrum. The design leverages birefringent titanium dioxide (TiO_2_) nanopillars to encode two distinct lateral phase profiles for orthogonal polarization states. By adjustment of the polarization direction of the transmitted light, multiple fringe patterns with controlled phase offsets can be selectively accessed. This enables phase-shifting functionality to be embedded into a single, static, and ultracompact optical element. Compared to existing approaches, this method offers high fringe fidelity and system stability while reducing the optical complexity of the system. Furthermore, we implemented this approach in a fringe projection profilometry system and demonstrated robust 3D surface reconstruction with a polarization-multiplexed three-step phase-shifting scheme. The underlying principle of polarization-controlled fringe encoding offers a compact and scalable strategy that can be applied to a broad range of optical imaging platforms.

The principle of polarization-controlled phase-shifting structured illumination is illustrated in Figure 1. The metasurface is designed to encode two phase grating profiles for *x*- and *y*-polarized light, which are structurally identical but laterally offset. This leads to polarization-dependent fringe patterns whose lateral phase shift can be tuned by adjusting the polarization of the transmitted light. For *x*-polarized light (θ = 0°), the transmitted electric field can be expressed as

1 $$E_{0{}^{\circ}}\left(x\right)=A\cos\left(kx+\phi \right)$$

yielding the intensity distribution

2 $$I_{0{}^{\circ}}\left(x\right)=\frac{A^{2}}{2}\left(1+\cos\left(2kx+2\phi \right)\right)$$

Similarly, for *y*-polarized light (θ = 90°)

3 $$E_{90{}^{\circ}}\left(x\right)=A\cos\left(kx-\phi \right),,I_{90{}^{\circ}}\left(x\right)=\frac{A^{2}}{2}\left(1+\cos\left(2kx-2\phi \right)\right)$$

For an arbitrary linear polarization angle, θ, the resulting field is a coherent superposition of the *x*- and *y*-polarized components, leading to an intensity profile

4 $$I_{\theta }\left(x\right)=A^{2}\left[\cos^{2}\left(\theta \right)\cos^{2}\left(kx+\phi \right)+\sin^{2}\left(\theta \right)\cos^{2}\left(kx-\phi \right)\right]$$

In particular, for θ = 45°, where both polarization components contribute equally, the transmitted field simplifies to

5 $$E_{45{}^{\circ}}\left(x\right)=\sqrt{2}A\cos\left(\phi \right)\cos\left(kx\right),,I_{45{}^{\circ}}\left(x\right)=A^{2}\cos^{2}\left(\phi \right)\left(1+\cos\left(2kx\right)\right)$$

This analysis shows that the polarization state of the light governs the lateral phase shift of the projected sinusoidal fringe pattern. In this work, we use polarization angles of 0°, 45°, and 90° to generate three fringe patterns with relative phase offsets of $-\frac{2\pi }{3}$ , 0, and $+\frac{2\pi }{3}$ , respectively. These patterns form a polarization-controlled phase-shifting sequence suitable for standard three-step phase retrieval. While a three-step configuration is demonstrated here, the concept is readily extendable to multistep phase-shifting schemes by appropriately selecting polarization states. The detailed derivation of the polarization-dependent intensity profiles is provided in Supporting Information S1. This polarization-encoded phase modulation principle underlies the metasurface design presented in this work.

**Figure 1:**
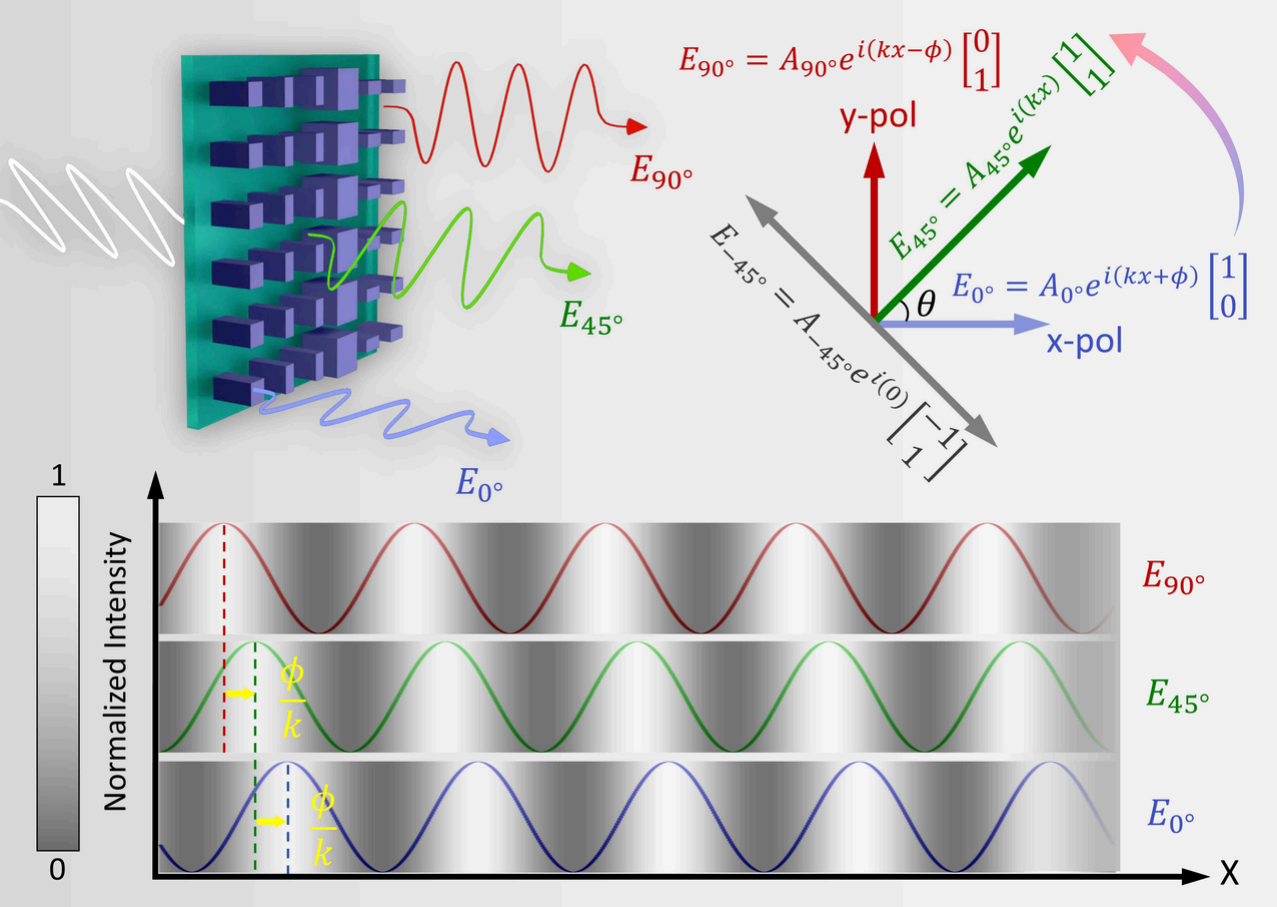
Schematic illustration of polarization-controlled phase-shifting structured illumination using a metasurface. The metasurface produces distinct fringe patterns with controlled lateral phase shifts, depending on the polarization state of the transmitted beam. This enables compact, multipattern structured light generation through polarization control.

To implement polarization-dependent phase modulation for structured light projection, we designed a dielectric metasurface composed of rectangular TiO_2_ nanopillars fabricated on a glass substrate, as shown in Figure 2a. Each nanopillar has a fixed height of 600 nm and is characterized by its width *W* and length *L*, independently influencing the phase delays imparted to *x*- and *y*-polarized light. The meta-atoms are arranged in a square lattice with a subwavelength period of 450 nm, enabling high-efficiency modulation. To guide the metasurface design, we numerically simulated the polarization-resolved transmission and phase responses of individual meta-atoms using finite element analysis at an operational wavelength of 532 nm. The resulting design library, shown in Figure 2b, maps the achievable phase shifts and transmission efficiencies as functions of the meta-atom dimensions under both *x*- and *y*-polarized illumination. This library serves as the basis for assigning geometries to implement polarization-dependent phase profiles across the metasurface.

**Figure 2:**
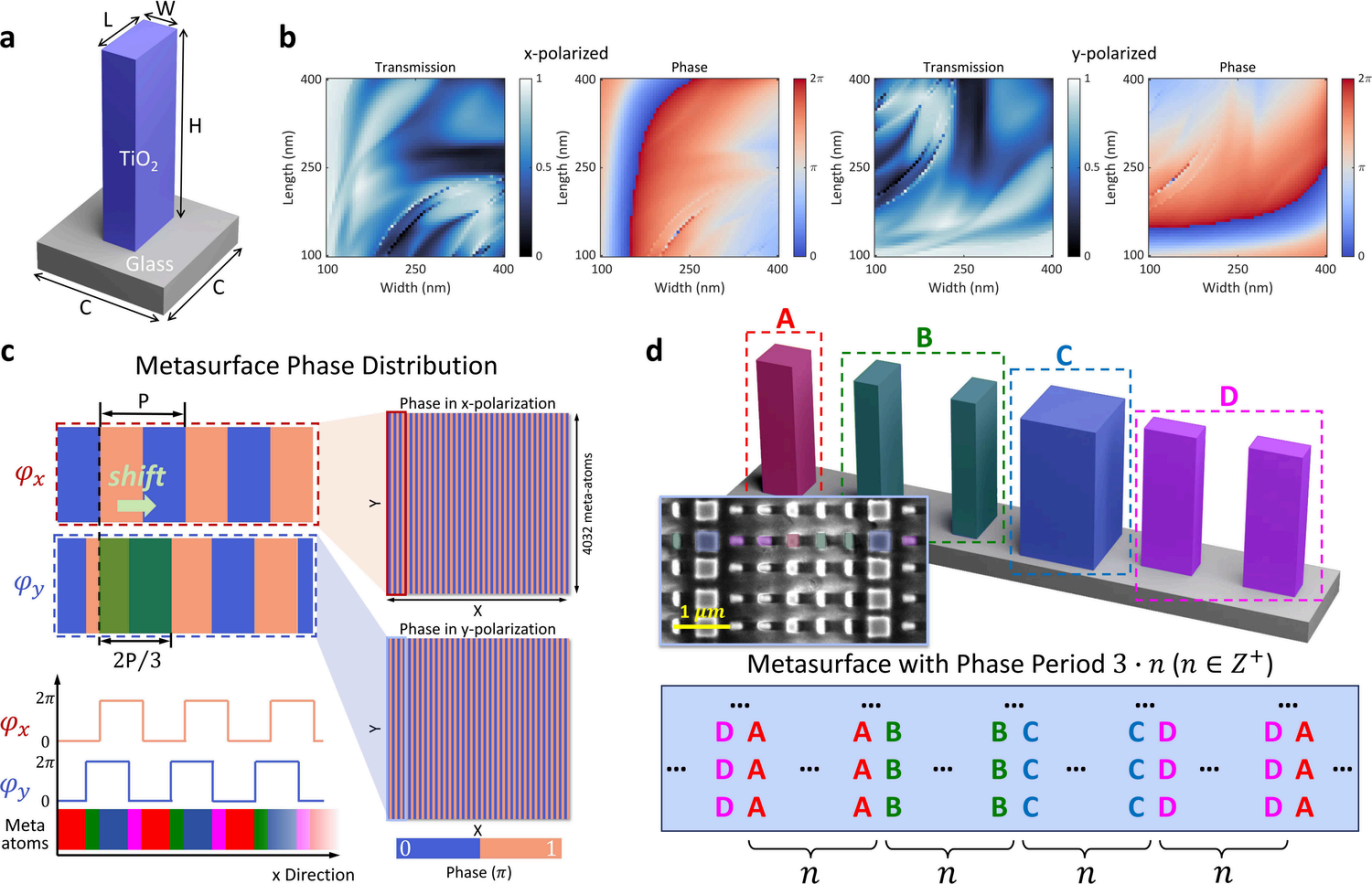
Metasurface design. (a) Schematic of TiO_2_ rectangular nanopillar meta-atoms forming the metasurface. (b) Simulated transmission and phase responses of meta-atoms under *x*- and *y*-polarized illumination as functions of pillar width and length. Simulations were performed using the finite-difference frequency-domain method in COMSOL Multiphysics 6.2. Periodic boundary conditions were applied laterally, and perfectly matched layers were used along the propagation direction. The refractive index of TiO_2_, measured by ellipsometry, was 2.47 (with negligible extinction) at a design wavelength of 532 nm; the substrate index was set to 1.53. (c) Polarization-dependent phase maps encoded into the metasurface, showing distinct grating profiles for *x*- and *y*-polarized incidence. (d) Structural layout of the metasurface, with an inset showing a scanning electron microscope image of a fabricated sample with *n* = 1.

Based on the simulated meta-atom response library, we designed a metasurface that simultaneously encodes two binary phase gratings for *x*- and *y*-polarized light, laterally offset by 2*P*/3, where *P* is the grating period (Figure 2c).^39,40^ These gratings generate sinusoidal fringe patterns with identical intensity profiles but lateral displacements that depend on the polarization state. The design is composed of six meta-atoms that implement the required discrete phase steps. Among them, only four have distinct geometries; the remaining two are structural duplicates used to complete the phase sequence. For clarity, these are grouped into four categories labeled A, B, C, and D. As illustrated in Figure 2d, the six-element phase unit is periodically repeated *n* times across the metasurface, yielding a total grating period of 3*n* pixels. In our implementation, we set *n* = 20, resulting in a total period of 60 pixels. Detailed optical responses of the four structural types are provided in Supporting Information S4.

The metasurface functions as a polarization-multiplexed diffractive element that modulates orthogonal polarization components independently. Under illumination by a linearly polarized plane wave with equal *x*- and *y*-polarized components, the encoded phase gratings generate a composite transmitted field comprising multiple spatial frequency components. At the metasurface plane (*z* = 0), the field can be described by a Fourier series

6 $$E_{\mathrm{trans}}\left(x,0\right)=\underset{m}{\sum }A_{m}e^{i\left(k+mK\right)x}$$

where *k* is the incident wavevector, *K* = 2π/*P* is the grating spatial frequency, and *A*_*m*_ are Fourier coefficients determined by the binary phase profile. Upon propagation, each diffraction order accumulates an additional phase

7 $$E\left(x,z\right)=\underset{m}{\sum }A_{m}e^{i\left(k+mK\right)x}e^{i\sqrt{k^{2}-{\left(k+mK\right)}^{2}}z}$$

Higher order components with |*mK*| > *k* are evanescent or strongly diverging and are effectively suppressed. At a finite distance, the observed field primarily consists of the fundamental modes

8 $$E_{\mathrm{obs}}\left(x,z\right)\approx A_{0}e^{ikx}+A_{1}e^{i\left(k+K\right)x}+A_{-1}e^{i\left(k-K\right)x}$$

which corresponds to a spatially modulated wave

9 $$E_{\mathrm{obs}}\left(x,z\right)\approx A\cos\left(Kx+\phi \right)$$

where *A* and ϕ depend on the amplitudes and relative phases of the retained components. The propagation behavior confirms that binary phase gratings, when appropriately designed, evolve into smooth sinusoidal patterns suitable for structured illumination. Crucially, the lateral phase shift between the polarization-encoded gratings is preserved in the transmitted field, providing a robust mechanism for the polarization-controlled fringe generation. This wavefront shaping capability underpins the phase-shifting illumination demonstrated in the experiments that follow. To implement this design, the metasurface was fabricated using standard electron-beam lithography followed by dry etching, as detailed in Supporting Information S6.

To evaluate the performance of the polarization-controlled metasurface, we experimentally characterized the quality of the generated phase-shifting structured illumination under different transmitted polarization states. This assessment focused on both the spatial fidelity of the projected fringe patterns and the accuracy of the retrieved phase information. The experimental setup is illustrated in Figure 3a. A collimated, linearly polarized laser beam with equal *x*- and *y*-polarized components was directed onto the metasurface by using a polarizer (P1). The resulting polarization-dependent phase-shifting fringe patterns were recorded at transmitted polarization angles of 0°, 45°, and 90°, selected via an analyzer (P2). Figure 3b shows the simulated fringe patterns corresponding to the three polarization angles, while Figure 3c presents the experimentally captured results. The lateral intensity profiles extracted from the experimental image closely match the simulated prediction (Figure 3d), confirming the fidelity of the metasurface in reproducing the designed fringe patterns. To retrieve the encoded phase information, we first normalized the recorded intensity patterns to account for the intensity scaling difference at 45° polarization. This ensures consistent phase retrieval, unaffected by global intensity variations. We then applied a standard three-step phase-shifting algorithm to the normalized fringe patterns. Given three phase-shifting fringe patterns *I*_0°_, *I*_45°_, and *I*_90°_, the wrapped phase ϕ­(*x*) is calculated as

10 $$\phi \left(x\right)=\tan^{-1}\left(\frac{\sqrt{3}\left(I_{0{}^{\circ}}-I_{90{}^{\circ}}\right)}{2I_{45{}^{\circ}}-I_{0{}^{\circ}}-I_{90{}^{\circ}}}\right)$$

The resulting wrapped phase distribution is shown in Figure 3e. A spatial phase unwrapping algorithm^41^ was then applied to recover a continuous phase map, as shown in Figure 3f. The smooth and well-defined phase profiles obtained from the measurements demonstrate the high optical quality and stability of the structured illumination produced by the metasurface, validating its effectiveness for precise and compact phase-shifting light projection.

**Figure 3:**
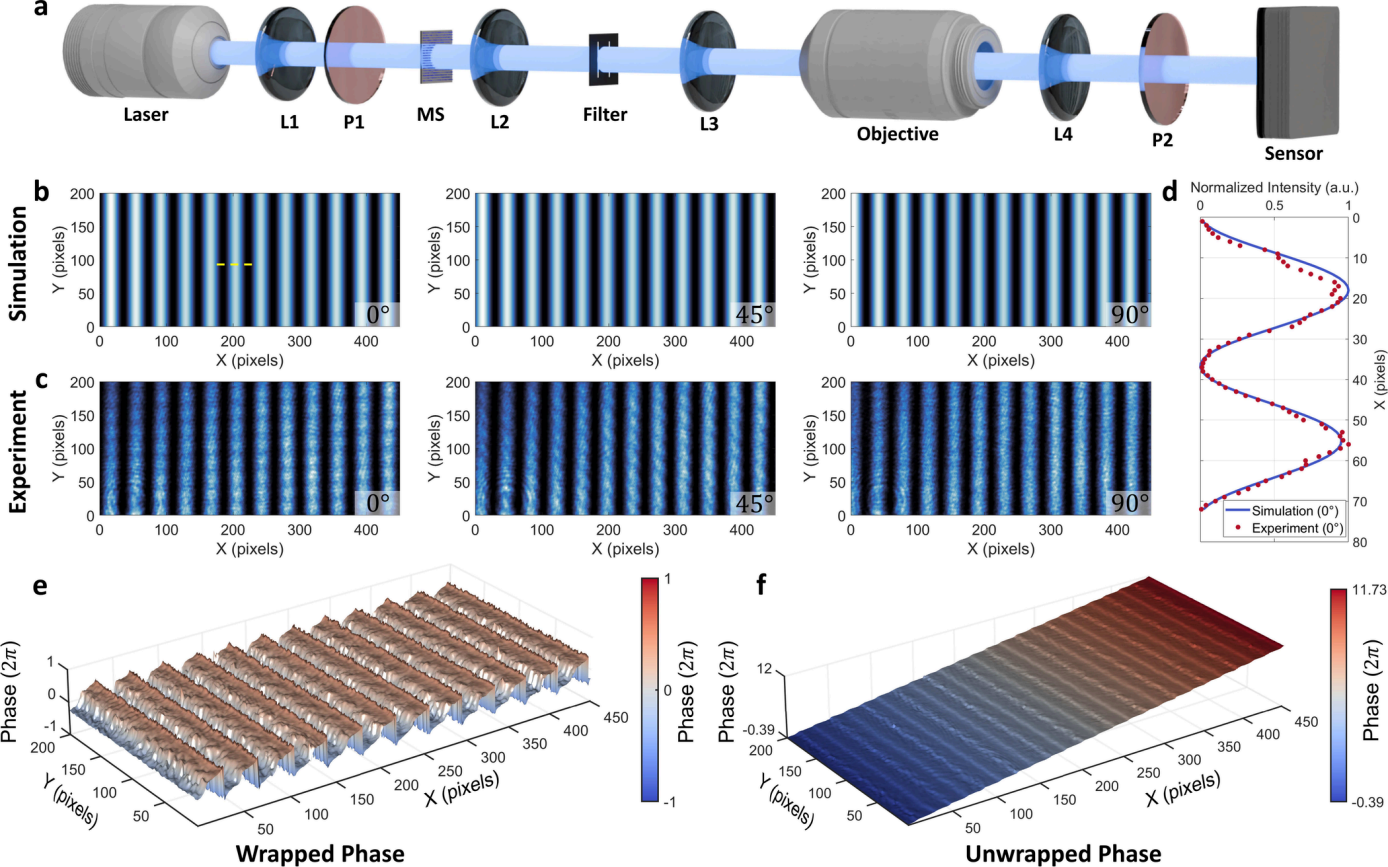
Characterization of polarization-controlled phase-shifting fringes. (a) Experimental setup for structured light generation and characterization. A 532 nm continuous-wave laser (WiTec, Oxford Instruments) was expanded and collimated using an achromatic lens (L1: AC254-050-A-ML, Thorlabs), followed by a linear polarizer (P1: LPVISE100-A, Thorlabs) to ensure equal *x*- and *y*-polarization components. The beam was incident on the metasurface and relayed through a 4*f* system (L2 and L3: AC254-075-AB-ML, Thorlabs) with a spatial filter placed in the Fourier plane. This configuration suppresses high-order diffraction and emulates free-space propagation within a compact optical path. The output was magnified by an objective (EC Epiplan-Neofluar 20×/0.5, ZEISS) and tube lens (L4: AC254-150-AB-ML, Thorlabs) and recorded with a scientific CMOS camera (ORCA-Fusion C14440-20UP, Hamamatsu). A second polarizer (P2) served as an analyzer to select the transmitted polarization state. (b) Simulated fringe patterns under polarization angles of 0°, 45°, and 90°. (c) Corresponding experimentally captured fringe patterns. (d) Comparison of simulated and measured lateral intensity profiles. (e) Wrapped phase retrieved from the three-step phase-shifted patterns in panel c. (f) Unwrapped phase distribution from panel e, showing a continuous phase profile.

To demonstrate the practical applicability of the metasurface-generated phase-shifting structured illumination, we integrated it into a fringe projection profilometry system for 3D surface measurement. The working principle and experimental setup are listed in Figure 4a. Polarization-controlled phase-shifting fringe patterns were projected onto the object surface and captured simultaneously by two cameras arranged in a calibrated stereo configuration.

**Figure 4:**
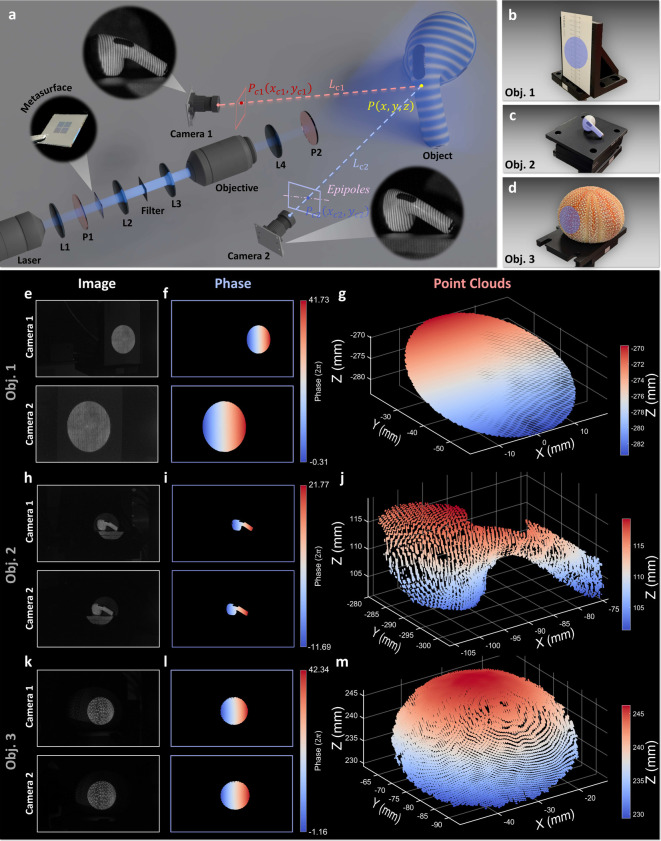
Fringe projection profilometry using metasurface-generated phase-shifting structured illumination. (a) Schematic illustration of the working principle and experimental setup for the 3D surface measurement. Phase-shifting structured light is projected by the metasurface and captured from two viewpoints using synchronized cameras (Basler acA1920-50gm, 16 mm focal length lenses). (b–d) Photographs of the test samples: a business card (Thorlabs), an earphone (AirPods Pro 2, Apple, Inc.), and a sea urchin shell. Shaded regions indicate the selected measurement areas. (e, h, and k) Captured structured illumination patterns at 0° polarization for the respective samples. (f, i, and l) Retrieved phase distributions from the corresponding patterns. (g, j, and m) Reconstructed 3D point clouds of the respective samples. All data analysis was performed using MATLAB R2022b.

The stereo system was calibrated using Zhang’s method,^42^ which estimates the intrinsic parameters of each camera as well as the relative rotation and translation between them.^43^ At each viewpoint, the local phase distribution was retrieved using a three-step phase-shifting algorithm,^40,44^ and these phase values served as discriminative descriptors for establishing pixel correspondences across the stereo views. Stereo rectification was applied to simplify the epipolar geometry, such that corresponding pixels lie along the same horizontal scanline.^43^ Let

11 $$P_{C1}=\left(u_{1},v\right),,P_{C2}=\left(u_{2},v\right)$$

denote the image coordinates of a 3D point *P* in cameras 1 and 2, respectively. The horizontal disparity is defined as

12 $$d=u_{1}-u_{2}$$

The perspective projection of a 3D point *P* = (*x*, *y*, *z*, 1)^T^ into image coordinates **x**_*i*_ = (*u*_*i*_, *v*_*i*_, 1)^T^ in camera *i* follows:

13 $$\lambda_{i}x_{i}=A_{i}\left[R_{i}t_{i}\right]P,,i\in \left\{1,2\right\}$$

where **A**_*i*_ is the intrinsic matrix and **R**_*i*_ and **t**_*i*_ are the extrinsic parameters of camera *i*. Scalar λ_*i*_ represents a projective scale factor. The relative pose between the two cameras is given by

14 $$R_{\mathrm{rel}}=R_{C2}R_{C1}^{T},,t_{\mathrm{rel}}=t_{C2}-R_{\mathrm{rel}}t_{C1}$$

Given a stereo pixel pair (**x**_1_, **x**_2_) matched via phase similarity, the corresponding 3D point is recovered by triangulation. Ideally, the viewing rays from both cameras intersect at a single point in the space. In practice, due to calibration uncertainties and noise, the 3D coordinates are computed by minimizing the squared distance to both rays

15 $$\underset{P}{\min}\left({∥P-L_{C1}∥}^{2}+{∥P-L_{C2}∥}^{2}\right)$$

where *L*_C1_ and *L*_C2_ represent the lines extending from the respective camera centers through the image points.^45,46^ Additional details on the stereo calculation and 3D reconstruction process are provided in Supporting Information S2 and S3.

Three representative objects with diverse geometries and surface properties, a business card, an earphone, and a sea urchin shell, were selected as test samples (Figure 4b–d). Polarization-controlled phase-shifting fringe patterns were projected onto each object and captured from two viewpoints using synchronized stereo cameras (Figure 4e, h, and k). To enhance the quality of the patterns, the captured images were processed by the frequency domain filtering to suppress noise.^47^ Phase distributions were then computed using a three-step phase-shifting algorithm (Figure 4f, i, and l), which served as the basis for establishing pixel correspondences across the stereo views. The region of interest for each object was extracted using a mask generated from the local modulation intensity of the structured light, ensuring that only areas with sufficient fringe contrast contributed to the 3D reconstruction.^48^

The reconstructed 3D point clouds (Figure 4g, j, and m) clearly capture the surface morphology and fine structural features of the test samples, demonstrating reliable performance across varying object geometries and material properties. These results validate the metasurface’s capability to support robust fringe projection for high-quality 3D surface measurement. By encoding polarization-dependent phase shifts into a static, compact optic, the system minimizes the optical complexity and footprint, offering a promising alternative to bulkier solutions that rely on dynamic spatial light modulation.

In conclusion, we have demonstrated a compact dielectric metasurface capable of generating polarization-controlled phase-shifting structured illumination. With the leverage of birefringent TiO_2_ nanopillars to encode distinct phase profiles for orthogonal polarizations, the metasurface enables tunable fringe pattern generation through simple polarization control. This static optical component integrates multiple phase-shifting states into a single planar layer without requiring active modulation or tunable elements, offering a robust and compact alternative to conventional structured light systems. We experimentally validated the metasurface in a fringe projection profilometry setup, achieving accurate 3D surface reconstruction of complex samples with diverse geometries and materials. The results highlight the metasurface’s capability to serve as a high-quality fringe pattern generator in a simplified optical architecture, dramatically reducing system size and alignment complexity. Beyond 3D profilometry, the demonstrated principle of polarization-encoded fringe modulation offers a flexible and compact solution for structured illumination across a variety of imaging modalities, including structured illumination microscopy,^6^ quantitative phase imaging,^49^ and digital holography.^50^ Its passive, scalable design and compatibility with polarization multiplexing make it particularly well-suited for integration into next-generation imaging and sensing platforms in biomedical diagnostics,^51^ industrial machine vision,^52^ and precision optical metrology.^53^

## Supplementary Material



## Notes

Mechanism of the polarization-controlled phase shift, 3D measurement setup calibration, mechanism of 3D imaging, meta-atom structures, quantitative validation of 3D reconstruction accuracy, and metasurface fabrication (PDF)
