# Supplementary material for: Phase-Shifting Structured Illumination with a Polarization-Encoded Metasurface
Source: Nano Lett. 2025 Jul 17;25(30):11696–702. doi: 10.1021/acs.nanolett.5c02789 (PMC12314901; doi:10.1021/acs.nanolett.5c02789)
Supplement: Supplementary file 1 [file nl5c02789_si_001.pdf]

Supporting Information for:
Phase-shifting structured illumination with polarization-encoded metasurface

Linzhi Yu,[†] Jesse Pietila,[†] Haobijam Johnson Singh,[†] and Humeyra Caglayan[*,†,‡]

†*Department of Physics, Tampere University, 33720, Tampere, Finland*

‡*Department of Electrical Engineering and Eindhoven Hendrik Casimir Institute, Eindhoven University of Technology, Eindhoven 5600 MB, The Netherlands*

E-mail: h.caglayan@tue.nl

S1: Mechanism of polarization-controlled phase shift

This section derives the intensity of the light field at any polarization angle θ. For the field at y-polarization ($\theta = 90°$):

$$E_{90^\circ} = A\cos(kx - \phi), \tag{S1}$$

and the corresponding intensity is given by:

$$I_{90^\circ} = |E_{90^\circ}|^2 = A^2\cos^2(kx - \phi) = \frac{A^2}{2}\left(1 + \cos(2kx - 2\phi)\right). \tag{S2}$$

For the field at x-polarization ($\theta = 0°$):

$$E_{0^\circ} = A\cos(kx + \phi), \tag{S3}$$

and the corresponding intensity is:

$$I_{0^\circ} = |E_{0^\circ}|^2 = A^2\cos^2(kx + \phi) = \frac{A^2}{2}\left(1 + \cos(2kx + 2\phi)\right). \tag{S4}$$

The electric field E_θ, polarized at an angle θ, is expressed as:

$$E_\theta = E_{90^\circ}\sin\theta + E_{0^\circ}\cos\theta. \tag{S5}$$

Substituting the expressions for E_{90° and E_{0°:

$$E_\theta = A\cos(kx - \phi)\sin\theta + A\cos(kx + \phi)\cos\theta, \tag{S6}$$

which simplifies to:

$$E_\theta = A\left[\cos(kx)\left(\cos(\phi)\cos(\theta) + \cos(\phi)\sin(\theta)\right) + \sin(kx)\left(\sin(\phi)\sin(\theta) - \sin(\phi)\cos(\theta)\right)\right]. \tag{S7}$$

Further simplification yields:

$$E_\theta = A\left[\cos(kx)\cos(\phi)(\cos\theta + \sin\theta) + \sin(kx)\sin(\phi)(\sin\theta - \cos\theta)\right]. \tag{S8}$$

Using trigonometric identities:

$$\cos\theta + \sin\theta = \sqrt{2}\cos(\theta - 45^\circ), \quad \cos\theta - \sin\theta = \sqrt{2}\sin(\theta - 45^\circ), \tag{S9}$$

we obtain:

$$E_\theta = \sqrt{2}A\left[\cos(kx)\cos(\phi)\cos(\theta - 45^\circ) + \sin(kx)\sin(\phi)\sin(\theta - 45^\circ)\right]. \tag{S10}$$

Defining $C_1 = \cos(\phi)\cos(\theta - 45^\circ)$ and $C_2 = \sin(\phi)\sin(\theta - 45^\circ)$, the field becomes:

$$E_\theta = \sqrt{2}A\left[C_1\cos(kx) + C_2\sin(kx)\right]. \tag{S11}$$

Expressing in cosine form:

$$E_\theta = \sqrt{2}A\sqrt{C_1^2 + C_2^2}\cos(kx - \delta), \tag{S12}$$

where $\delta = \tan^{-1}\left(\frac{\sin(\phi)\sin(\theta-45^\circ)}{\cos(\phi)\cos(\theta-45^\circ)}\right)$. The corresponding intensity is:

$$I_\theta = |E_\theta|^2 = 2A^2\left[\cos^2(\phi)\cos^2(\theta - 45^\circ) + \sin^2(\phi)\sin^2(\theta - 45^\circ)\right](1 + \cos(2kx - 2\delta)). \tag{S13}$$

For $\theta = 45^\circ$, the electric field reduces to:

$$E_{45^\circ} = \sqrt{2}A\cos(\phi)\cos(kx), \tag{S14}$$

with intensity:

$$I_{45^\circ} = A^2\cos^2(\phi)\left(1 + \cos(2kx)\right). \tag{S15}$$

Thus, for I_{90°, the phase is -2ϕ, corresponding to a lateral shift:

$$\Delta x_{90^\circ} = \frac{-2\phi}{2k} = \frac{-\phi}{k}. \tag{S16}$$

For I_{0°, the phase is 2ϕ, corresponding to a lateral shift:

$$\Delta x_{0^\circ} = \frac{2\phi}{2k} = \frac{\phi}{k}. \tag{S17}$$

For I_{45°, the phase is 0, corresponding to a lateral shift:

$$\Delta x_{45^\circ} = 0. \tag{S18}$$

S2: 3D measurement setup calibration

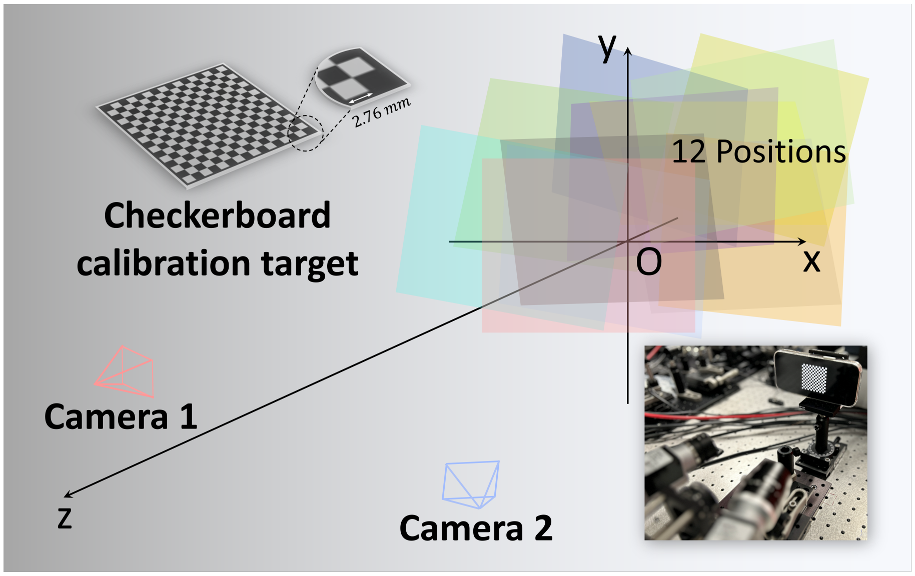

Figure S1: Camera calibration using Zhang's method. The inset shows the experimental setup used for calibration.

In this paper, the experiment adopts Zhang's camera calibration method[1,2] to estimate both the intrinsic parameters (such as focal lengths, principal point, and skew), the extrinsic parameters (rotation and translation between the camera and world coordinates), and the distortion coefficients of each camera in the stereo system. This method requires only a planar calibration pattern observed from multiple poses, making it efficient and well-suited for our setup. As shown in Figure S1, a binocular stereo system was calibrated using a planar checkerboard pattern displayed on a mobile phone (iPhone 16 Pro, Apple Inc.). The target featured a checkerboard pattern with a square size of 2.76 mm. A total of 12 images were captured from varying poses to provide sufficient geometric constraints. The calibration was performed separately for each camera, allowing recovery of the intrinsic parameters for both, as well as the relative pose between them.

The imaging process is modeled by the perspective projection equation:

$$\lambda \mathbf{x} = \mathbf{A}[\mathbf{R}\ \mathbf{t}]\,\mathbf{X}, \tag{S19}$$

where

$$\mathbf{x} = \begin{bmatrix} u \\ v \\ 1 \end{bmatrix}, \quad \mathbf{X} = \begin{bmatrix} X \\ Y \\ Z \\ 1 \end{bmatrix},$$

λ is a scale factor, and

$$\mathbf{A} = \begin{bmatrix} f_x & \gamma & u_0 \\ 0 & f_y & v_0 \\ 0 & 0 & 1 \end{bmatrix} \tag{S20}$$

is the intrinsic matrix. The extrinsic parameters are represented by the rotation matrix $\mathbf{R}$ and the translation vector $\mathbf{t}$. In addition to the intrinsic and extrinsic parameters, Zhang's method also estimates the distortion coefficients that model lens imperfections. Let (x, y) denote the normalized image coordinates after applying the perspective projection:

$$x = \frac{X_c}{Z_c}, \quad y = \frac{Y_c}{Z_c},$$

where (X_c, Y_c, Z_c) is the three-dimensional (3D) point in the camera coordinate frame. The distorted image coordinates (x_d, y_d) are given by:

$$x_d = x\left(1 + k_1 r^2 + k_2 r^4 + k_3 r^6\right) + 2p_1 xy + p_2(r^2 + 2x^2), \tag{S21}$$

$$y_d = y\left(1 + k_1 r^2 + k_2 r^4 + k_3 r^6\right) + 2p_2 xy + p_1(r^2 + 2y^2), \tag{S22}$$

where $r^2 = x^2 + y^2$, k_1, k_2, k_3 are the radial distortion coefficients, and p_1, p_2 are the tangential distortion coefficients. These distortion-corrected coordinates are then mapped into pixel coordinates using the intrinsic matrix $\mathbf{A}$. Because the calibration target is planar, we set

$Z = 0$, reducing the projection to a 2D homography:

$$\lambda \mathbf{x} = \mathbf{A} \begin{bmatrix} \mathbf{r}_1 & \mathbf{r}_2 & \mathbf{t} \end{bmatrix} \begin{bmatrix} X \\ Y \\ 1 \end{bmatrix}, \tag{S23}$$

where $\mathbf{r}_1$ and $\mathbf{r}_2$ are the first two columns of the rotation matrix $\mathbf{R}$, and the resulting homography is:

$$\mathbf{H} = \mathbf{A} \begin{bmatrix} \mathbf{r}_1 & \mathbf{r}_2 & \mathbf{t} \end{bmatrix}. \tag{S24}$$

Multiple views yield a set of homographies $\{\mathbf{H}_i\}$, from which the intrinsic matrix $\mathbf{A}$ is estimated by solving a system of linear constraints. The rotation matrix $\mathbf{R}$ is composed of three orthonormal column vectors:

$$\mathbf{R} = \begin{bmatrix} \mathbf{r}_1 & \mathbf{r}_2 & \mathbf{r}_3 \end{bmatrix}, \tag{S25}$$

where $\mathbf{r}_1$ and $\mathbf{r}_2$ are estimated from the homography decomposition, and $\mathbf{r}_3$ is recovered by enforcing the orthonormality constraint $\mathbf{r}_3 = \mathbf{r}_1 \times \mathbf{r}_2$. The extrinsic parameters for each view are then recovered by decomposing the homography:

$$\mathbf{r}_1 = \frac{\mathbf{A}^{-1}\mathbf{h}_1}{\|\mathbf{A}^{-1}\mathbf{h}_1\|}, \tag{S26}$$

$$\mathbf{r}_2 = \frac{\mathbf{A}^{-1}\mathbf{h}_2}{\|\mathbf{A}^{-1}\mathbf{h}_2\|}, \tag{S27}$$

$$\mathbf{t} = \frac{\mathbf{A}^{-1}\mathbf{h}_3}{\|\mathbf{A}^{-1}\mathbf{h}_1\|}, \tag{S28}$$

where $\mathbf{h}_1, \mathbf{h}_2, \mathbf{h}_3$ are the columns of $\mathbf{H}$.

S3: Mechanism of 3D imaging

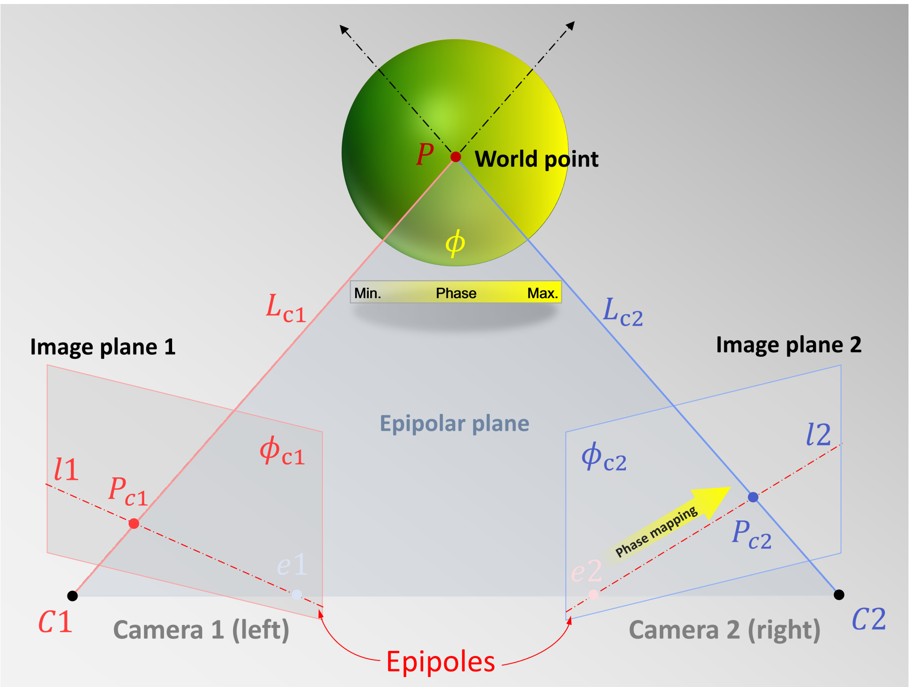

Figure S2: Schematic illustration of binocular stereo vision and phase-shifting structured illumination for 3D surface measurement.

To perform 3D reconstruction, a conventional binocular stereo vision framework is employed.[2–4] Figure S2 illustrates the reconstruction process and associated geometry. Once the intrinsic parameters of each camera and their relative pose are determined via Zhang's calibration method, the 3D coordinates of a scene point can be recovered through triangulation. In our experiment, each pixel in the left and right views is associated with an unwrapped phase value, denoted ϕ_{C1} and ϕ_{C2}, respectively. These phase values serve as robust descriptors for establishing correspondences along epipolar lines. After stereo rectification, the epipolar geometry simplifies such that corresponding points lie on the same horizontal scanline. Let

$$P_{C1} = (u_1, v_1), \quad P_{C2} = (u_2, v_2) \tag{S29}$$

denote the image coordinates of a world point P in the left ($C1$) and right ($C2$) views. After

rectification, the vertical coordinates are aligned:

$$v_1 = v_2, \tag{S30}$$

and the horizontal disparity is defined as

$$d = u_1 - u_2. \tag{S31}$$

Phase similarity is used as the matching criterion. For each valid pixel (u_1, v) in the left image with phase $\phi_{C1}(u_1, v)$, we search along the epipolar line in the right image to find the pixel (u_2, v) whose phase $\phi_{C2}(u_2, v)$ best matches ϕ_{C1}. This yields a pixel pair (P_{C1}, P_{C2}) assumed to correspond to the same 3D world point. Given the calibrated stereo system, the projection of a 3D point P into each camera is modeled by the perspective projection equation:

$$\lambda_i \, \mathbf{x}_i = \mathbf{A}_i [\mathbf{R}_i \; \mathbf{t}_i] \, \mathbf{P}, \quad i \in \{C1, C2\}, \tag{S32}$$

where $\mathbf{x}_{C1} = \begin{bmatrix} u_1 \\ v_1 \\ 1 \end{bmatrix}$, $\mathbf{x}_{C2} = \begin{bmatrix} u_2 \\ v_2 \\ 1 \end{bmatrix}$, and $\mathbf{A}_{C1}, \mathbf{A}_{C2}$ are the intrinsic matrices of the two cameras. The relative pose between the cameras is computed as:

$$\mathbf{R}_{\text{rel}} = \mathbf{R}_{C2} \mathbf{R}_{C1}^{\top}, \quad \mathbf{t}_{\text{rel}} = \mathbf{t}_{C2} - \mathbf{R}_{\text{rel}} \, \mathbf{t}_{C1}. \tag{S33}$$

Let $C1$ and $C2$ denote the optical centers of the two cameras. The rays corresponding to image projections P_{C1} and P_{C2} are defined as lines extending from $C1$ and $C2$ through the respective image points. In an ideal, noise-free scenario, these rays intersect at the true 3D point P. In practice, due to calibration and matching errors, the rays may not intersect exactly. Triangulation is therefore performed by finding the point P that minimizes its

distance to both rays:

$$\min_{P} \left(\|P - L_{C1}\|^2 + \|P - L_{C2}\|^2 \right), \tag{S34}$$

where L_{C1} and L_{C2} denote the lines (rays) extending from the camera centers through the matched image coordinates. This process, combining phase-based disparity estimation with stereo calibration, enables accurate recovery of 3D surface geometry.

S4: Meta-atom structures

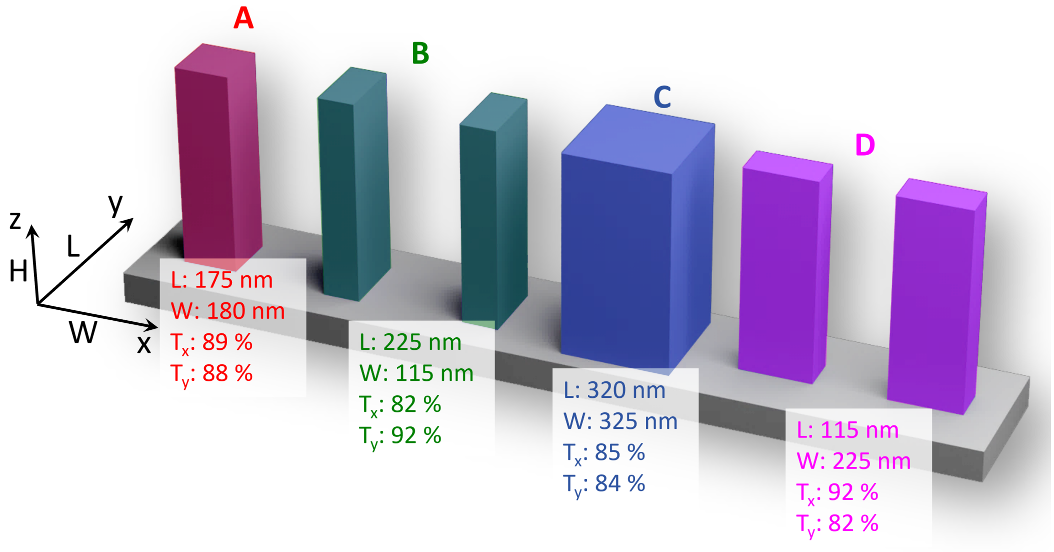

Figure S3: Structural parameters and transmission efficiencies of the meta-atoms used in the metasurface design. L and W represent the length and width of the meta-atoms, respectively. T_x and T_y denote the transmission efficiencies under x- and y-polarized illumination.

The structural parameters of the meta-atoms used in this study are shown in Figure S3. The transmission efficiencies under x- and y-polarized illumination are denoted by T_x and T_y, respectively. The metasurface comprises four distinct meta-atom designs, grouped as A, B, C, and D. Among these, groups B and D are supercells, each composed of two individual meta-atoms. The meta-atoms were carefully designed to provide the required phase distribution while maintaining high transmission efficiency, thereby supporting the intended metasurface functionality.

Figure S4 presents the simulated electric and magnetic field energy distributions within the meta-atoms under linearly polarized illumination at a wavelength of 532 nm. The strong field confinement observed in each structure confirms their ability to achieve localized and independent phase modulation, enabling precise wavefront control across the metasurface.

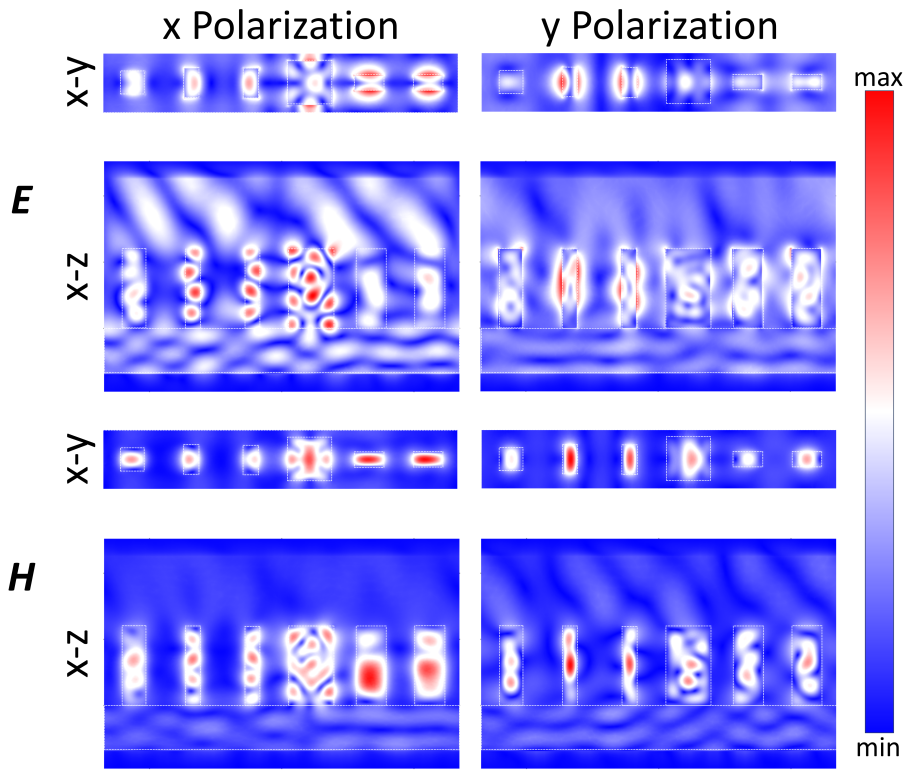

Figure S4: Normalized electric and magnetic field distributions of the meta-atoms under linearly polarized illumination at a wavelength of 532 nm.

S5: Quantitative validation of 3D reconstruction accuracy

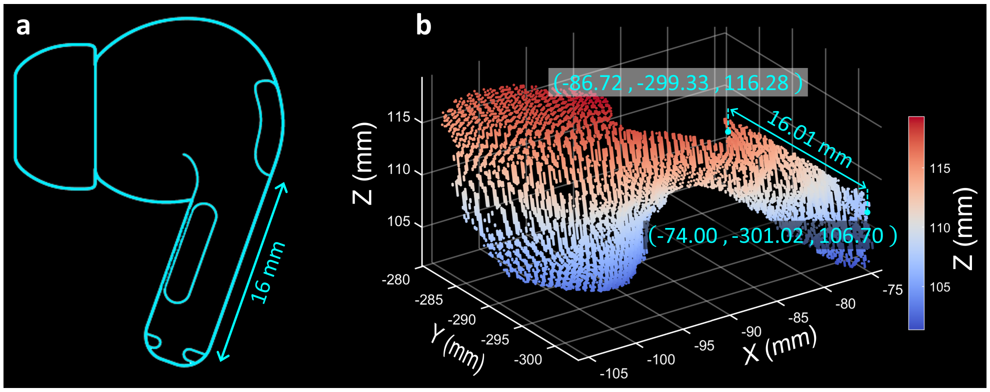

Figure S5: Comparison of the distance between the microphone opening and the charging anode of the earphone. (a) Specified reference distance: 16.00 mm. (b) Distance measured from the reconstructed 3D point cloud: 16.01 mm.

To quantitatively validate the 3D reconstruction, we compared a key dimension of the earphone sample with its specified reference value. The distance between the microphone opening and the charging anode is 16.00 mm as shown in Figure S5a. The corresponding distance measured from the reconstructed 3D point cloud is 16.01 mm, as shown in Figure S5b. This close match demonstrates the high accuracy of our system.

S6: Metasurface fabrication

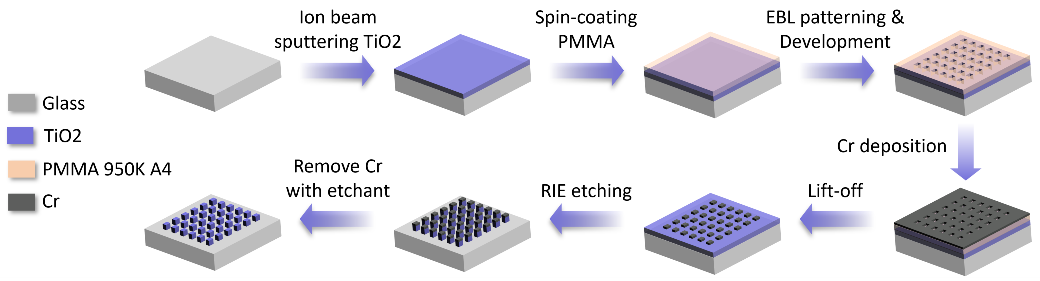

Figure S6: Schematic illustration of the metasurface fabrication process.

The fabrication process of the metasurface is illustrated in Figure S6.[5] The process began with depositing a 600 nm titanium dioxide (TiO_2) layer onto a 400 µm thick glass substrate (Präzisions Glas & Optik GmbH) via ion beam sputtering (Cutting Edge Coatings Navigator 700). A 250 nm polymethyl methacrylate (PMMA) 950K A4 electron-beam resist was then spin-coated onto the substrate and baked at 180 °C for 90 seconds. To reduce charge accumulation and facilitate substrate alignment during electron-beam lithography, a 20 nm aluminum (Al) conductive layer was deposited via e-beam evaporation (Instrumentti Mattila IM-9912). The resist was patterned using a pre-designed layout with an electron-beam lithography system (Vistec EPBG5000pES). After exposure, the Al was removed using an AZ351B:deionized water solution (1:5), and the resist was developed in a methyl isobutyl ketone:isopropanol (MIBK:IPA, 1:3) solution. A 50 nm chromium (Cr) hard mask was deposited through e-beam evaporation, and pattern transfer was accomplished via an overnight lift-off process in Remover 1165. The TiO_2 layer was etched using reactive ion etching (Oxford Plasmalab System 100) with a gas mixture of sulfur hexafluoride (SF_6, 5 sccm) and argon (Ar, 15 sccm) at 125 W, 30 mTorr, and 21 °C. Finally, the Cr hard mask was removed with a chromium etchant (Sigma-Aldrich).

References

(1) Zhang, Z. A flexible new technique for camera calibration. *IEEE Transactions on Pattern Analysis and Machine Intelligence* **2002**, *22*, 1330–1334.

(2) Bradski, G.; Kaehler, A. *Learning OpenCV: Computer Vision with the OpenCV Library*; O'Reilly Media: USA, 2008.

(3) Hartley, R.; Zisserman, A. *Multiple View Geometry in Computer Vision*, 2nd ed.; Cambridge University Press: USA, 2003.

(4) Salvi, J.; Fernandez, S.; Pribanic, T.; Llado, X. A state of the art in structured light patterns for surface profilometry. *Pattern Recognition* **2010**, *43*, 2666–2680.

(5) Yang, W.; Zhou, J.; Tsai, D. P.; Xiao, S. Advanced manufacturing of dielectric metadevices. *Photonics Insights* **2024**, *3*, R04–R04.
